# Role of Keap1-Nrf2 Signaling in Anhedonia Symptoms in a Rat Model of Chronic Neuropathic Pain: Improvement With Sulforaphane

**DOI:** 10.3389/fphar.2018.00887

**Published:** 2018-08-08

**Authors:** Shan Li, Chun Yang, Xi Fang, Gaofeng Zhan, Niannian Huang, Jie Gao, Hui Xu, Kenji Hashimoto, Ailin Luo

**Affiliations:** ^1^Department of Anesthesiology, Tongji Hospital, Tongji Medical College, Huazhong University of Science and Technology, Wuhan, China; ^2^Division of Clinical Neuroscience, Center for Forensic Mental Health, Chiba University, Chiba, Japan

**Keywords:** pain, anhedonia, sulforaphane, Keap1-Nrf2, medial prefrontal cortex, hippocampus, spinal cord, skeletal muscle

## Abstract

Patients with chronic neuropathic pain frequently suffer from symptoms of anhedonia (loss of pleasure), which is a core clinical manifestation of depression. Accumulating studies have shown the beneficial effects of the natural compound sulforaphane (SFN), an activator of nuclear factor (erythroid-derived 2)-like 2 (Nrf2), on depression-like phenotype through a potent anti-inflammatory effect. However, it is unknown whether SFN confers beneficial effects in neuropathic pain-associated anhedonia. Spared nerve injury (SNI) is classical rodent model of chronic neuropathic pain. We here used a rat model of SNI. Hierarchical cluster analysis of sucrose preference test (SPT) results was used to classify the SNI rats with or without an anhedonia phenotype. Nrf2 protein expression was significantly decreased in the medial prefrontal cortex (mPFC), hippocampus, spinal cord, and skeletal muscle, but not in the nucleus accumbens, in anhedonia-susceptible rats compared with sham or anhedonia-resistant rats. The expression of Kelch-like erythroid cell-derived protein with CNC homology (ECH)-associated protein 1 (Keap1), a partner of Nrf2, in mPFC, hippocampus, and muscle of anhedonia-susceptible rats was also significantly lower than that in sham or anhedonia-resilient rats. Subsequent SFN administration after SNI surgery exerted therapeutic effects on reduced mechanical withdrawal threshold (MWT) scores, but not on sucrose preference, through the normalization of Keap1-Nrf2 signaling in the spinal cords of anhedonia-susceptible rats. Interestingly, treatment with SFN 30 min prior to SNI surgery significantly attenuated reduced MWT scores and sucrose preference, and restored tissue Keap1 and Nrf2 levels. In conclusion, this study suggests that decreased Keap1-Nrf2 signaling in mPFC, hippocampus, and muscle may contribute to anhedonia susceptibility post-SNI surgery, and that SFN exerts beneficial effects in SNI rats by normalization of decreased Keap1-Nrf2 signaling.

## Introduction

Patients with chronic pain often suffer with depressive symptoms. Previous clinical studies have demonstrated that the incidence of comorbid chronic pain and depression is approximately 30 to 50% (Bair et al., 2003; Gustorff et al., 2008; Radat et al., 2013). Thus, comorbid pain and depression are a serious clinical, social, and economic issue that needs to be resolved. However, the underlying mechanisms and therapeutic strategies for managing this comorbidity remain undetermined.

Non-steroidal anti-inflammatory drugs (NSAIDs) and opioid drugs are widely used for pain relief. However, several studies have shown that NSAIDs are not effective in approximately half of patients with chronic pain (White, 2005; Vanegas et al., 2010; Shah and Mehta, 2012). Furthermore, although opioids have powerful analgesic effects, their considerable side effects limit their widespread use when administered in larger doses (Sehgal et al., 2013). Opioid crisis and drug overdose-related deaths are a serious problem in the United States (Volkow and Collins, 2017). New treatment options for opioid-use disorders are sorely needed. Although analgesics and antidepressant agents are currently prescribed for depression in patients with somatic symptoms or chronic pain, drugs without significant side effects are needed for treating the comorbidity of pain and depression.

Nuclear factor (erythroid-derived 2)-like 2 (Nrf2) is a transcription factor that plays a central role in cellular defense against oxidative and electrophilic insults (Kobayashi et al., 2013; Ma, 2013; Suzuki et al., 2013; Suzuki and Yamamoto, 2015; Cuadrado et al., 2018; Yamamoto et al., 2018). Under normal conditions, Nrf2 is repressed by Kelch-like erythroid cell-derived protein with CNC homology (ECH)-associated protein 1 (Keap1), which is an adaptor protein for Nrf2 degradation (Suzuki et al., 2013; Suzuki and Yamamoto, 2015; Yamamoto et al., 2018). During oxidative stress, including inflammation, Nrf2 is derepressed, which activates the transcription of cytoprotective genes. In addition, the Keap1-Nrf2 system is also involved in attenuating inflammation-associated pathogenesis (Kobayashi et al., 2013; Suzuki et al., 2013; O’Connell and Hayes, 2015; Suzuki and Yamamoto, 2015; Yamamoto et al., 2018). In the learned helplessness (LH) paradigm, Keap1 and Nrf2 protein levels in the prefrontal cortex and dentate gyrus of the hippocampus in LH (susceptible) rats were lower than those in control and non-LH (resilient) rats (Zhang et al., 2018). Furthermore, the expression of Keap1 and Nrf2 proteins in the parietal cortex of depressed patients was lower than that in controls, suggesting that Keap1-Nrf2 signaling contributes to stress resilience, which plays a key role in the pathophysiology of depression (Zhang et al., 2018).

Nrf2 signaling pathway has been commonly reported to be activated by food supplements and natural compounds (Bhaskaran et al., 2013; Menghini et al., 2016, 2018; Efentakis et al., 2017). Sulforaphane (SFN), a natural potent anti-inflammatory compound, is an organosulfur compound derived from glucoraphanin (a glucosinolate precursor of SFN) which is fully enriched in cruciferous vegetables (Zhang et al., 1992; Fahey et al., 1997; Dinkova-Kostova et al., 2017; Fahey et al., 2017). SFN is reported to have potent anti-inflammatory effects through the activation of Nrf2. Previously, we reported that pretreatment with SFN has prophylactic effects on depression-like phenotype and dendritic spine changes in an inflammation-induced model of depression (Zhang et al., 2017), and that novel Nrf2 activators (TBE-31 and MCE-1) demonstrate antidepressant effects in an inflammation model of depression (Yao et al., 2016b). Furthermore, dietary intake of glucoraphanin during juvenile and adolescent stages confers resilience to chronic social defeat stress in adulthood (Yao et al., 2016b). Previous reports have shown that intrathecal administration of SFN attenuated mechanical allodynia and thermal hyperalgesia in spinal nerve transfection-injured mice (Kim et al., 2010), and that SFN inhibited complete Freund’s adjuvant-induced allodynia and hyperalgesia (Redondo et al., 2017). Taken together, it is likely that SFN is a potential natural compound for treating comorbid pain and depression.

The purpose of the present study was to examine the role of the Keap1-Nrf2 signaling system in selected tissues following surgery for spared nerve injury (SNI). Furthermore, we investigated whether abnormalities in behaviors and Keap1-Nrf2 levels in selected tissues following SNI are attenuated with subsequent administration of SFN. Finally, we examined whether pretreatment with SFN could prevent pain and anhedonia-like symptoms and alter Keap1-Nrf2 protein expression in selected tissues following SNI.

## Materials and Methods

### Animals

Male Sprague Dawley (SD) rats (weighing 180–230 g) were purchased from the Laboratory Animal Centre of Tongji Medical College, Huazhong University of Science and Technology (Wuhan, China). A total of 98 rats were enrolled and were divided into groups in accordance with the random number table. The animals were housed under 12 h light/dark cycle with free access to food and water. Procedures of this animal experiment were in accordance with the National Institute of Health Guide for the Care and Use of Laboratory Animals. The experimental protocols were approved by the Experimental Animal Committee of Tongji Hospital, Tongji Medical College, Huazhong University of Science and Technology (No. 34724466).

### Experimental Design

**FIGURE 1:**
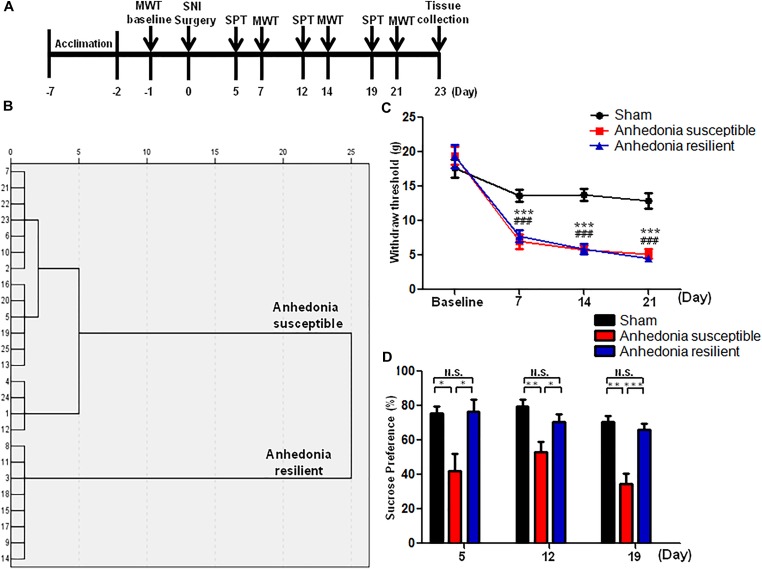
The results of MWT and SPT in sham, anhedonia-susceptible, and anhedonia-resilient rats. **(A)** The schedule of the experiment. SNI surgery was performed on day 0 after acclimation. MWT was measured on day 7, 14, and 21 after SNI, respectively. SPT was performed on day 5, 12, and 19 after SNI, respectively. **(B)** Dendrogram of hierarchical clustering analysis. A total of 25 SNI rats were divided into anhedonia-susceptible and anhedonia-resilient groups by SPT results. **(C)** MWT [Time: *F*_(3,21)_ = 38.352_,_ *P* < 0.001; Group: *F*_(2,14)_ = 66.232_,_ *P* < 0.001; Interaction: *F*_(6,42)_ = 9.711_,_ *P* < 0.001] was measured on day 7, 14, and 21 in the sham, anhedonia-resilient, and anhedonia-susceptible groups after SNI, respectively. Data are shown as mean ± SEM (*n* = 8). ^∗∗∗^*P* < 0.001, susceptible group vs. sham group; ^###^*P* < 0.001, resilient group vs. sham group. **(D)** SPT [Time: *F*_(2,14)_ = 2.523_,_ *P* = 0.13; Group: *F*_(2,14)_ = 30.388_,_ *P* < 0.001; Interaction: *F*_(4,28)_ = 0.687_,_ *P* = 0.609] was measured in the sham, anhedonia-resilient, and anhedonia-susceptible groups on day 5, 12, and 19 after SNI, respectively. Data are shown as mean ± SEM (*n* = 8). ^∗^*P* < 0.05, ^∗∗^*P* < 0.01, or ^∗∗∗^*P* < 0.001. MWT, mechanical withdrawal test; N.S., not significant; SNI, spared nerve injury; SPT, sucrose preference test.

As shown in **Figure 1A**, rats were acclimated to environment for 6 days. Then the mechanical withdrawal threshold (MWT) was performed 1 day before the SNI surgery for baseline measurement. MWT and sucrose preference test (SPT) were implemented from day 2 to 5, 9 to 12, and 16 to 19 after surgery, respectively. Twenty-three days after SNI surgery, medial prefrontal cortex (mPFC), hippocampus, and nucleus accumbens (NAc) of brain, L2-5 spinal cord, skeletal muscle, and liver were collected. Tissue samples were stored at -80°C before Western blot analysis. A single dose of SFN (30 mg/kg, Absin Bioscience Inc., Shanghai, China) was intraperitoneally injected before or after SNI surgery to investigate its effects on pain and anhedonia symptoms (**Figures 3A, 5A**). The dose (30 mg/kg) of SFN was used as previously reported (Shirai et al., 2012, 2015; Zhang et al., 2017).

### SNI

The SNI surgery was performed as previously described (Fang et al., 2018). Rats were anesthetized with 10% chloral hydrate (3 ml/kg) and then the skin of left thigh was incised. The sciatic nerve and its three terminal branches after bluntly dissecting biceps femoris muscle were totally exposed. The common peroneal and tibial nerves were ligated with a 4-0 silk and cut off the distal to the ligation. The muscle and skin were sutured with a 4-0 silk. Rats in the sham group were exposed to the sciatic nerve and its three terminal branches but without ligated and cut off the common peroneal and tibial nerves.

### MWT

Before MWT, rats were placed in plexiglass chambers with a wire net floor for 30 min avoiding the stress resulting from the test conditions. The Electronic Von Frey (UGO BASILE S.R.L., Italy) filaments were applied to the lateral 1/3 of right paws. The paws quick withdrawal or flinching was considered as a positive response. Every filament stimuli were applied 4 times with a period of 30 s interval (Fang et al., 2018).

### SPT

Rats were exposed to water and 1% sucrose solution for 48 h, followed by 24 h of water and food deprivation and a 24 h exposure to two identical bottles, one is water, and another is 1% sucrose solution. The bottles containing water and sucrose were weighed before and at the end of this period and the sucrose preference was determined (Fang et al., 2018).

### Western Blot

Samples were homogenized with RIPA buffer (150 mM sodium chloride, Triton X-100, 0.5% sodium deoxycholate, 0.1% sodium dodecyl sulfate, 50 mM Tris, pH 8.0) at 4°C for 30 min, then were centrifuged for 15 min at 4°C. BCA protein assay kit (Boster, Wuhan, China) was used to determine the protein levels in supernatant. The samples were separated by 10% sodium dodecyl sulfate-polyacrylamide gel electrophoresis and were transferred to polyvinylidene fluoride membranes (Millipore, Bedford, MA, United States). Bands were blocked with 5% BSA in TBST (0.1% Tween 20 in Tris-buffered saline) for 1 h at room temperature. Relative primary antibodies were incubated at 4°C overnight: rabbit Keap1 (1:1,000, Affinity, Cincinnati, OH, United States), rabbit Nrf2 (1:1,000; Affinity, Cincinnati, OH, United States), and mouse GAPDH (1:1,000, Qidongzi, Wuhan, China). Then bands were washed with TBST and incubated second antibody for 2 h at room temperature: goat anti-rabbit IgG horseradish peroxidase or goat anti-mouse IgG horseradish peroxidase (1:5,000, Qidongzi, Wuhan, China). Finally, these bands were detected by enhanced chemiluminescence reagents (Qidongzi, Wuhan, China) with the ChemiDoc XRS chemiluminescence imaging system (Bio-Rad, Hercules, CA, United States).

### Statistical Analyses

The data show as the mean ± standard error of the mean (SEM). Analysis was performed using PASW Statistics 20 (formerly SPSS Statistics; SPSS). Comparisons between groups were performed using the one-way analysis of variance (ANOVA) or two-way ANOVA, followed by *post hoc* Tukey test. In Hierarchical cluster analysis, the data were firstly standardized by *z*-scores. Then, hierarchical cluster analysis of SPT results was performed using Ward’s method and applying squared Euclidean distance as the distance measure, and mice were classified as anhedonia-susceptible rats or anhedonia-resilient rats (Fang et al., 2018). The *P*-values of less than 0.05 were considered statistically significant.

## Results

### Comparison of MWT and SPT Among the Sham, Anhedonia-Susceptible and Anhedonia-Resilient Rats

A total of 25 SNI rats were divided into anhedonia-susceptible and anhedonia-resilient groups by hierarchical clustering analysis of SPT results (**Figure 1B**). MWT was significantly decreased in both anhedonia-susceptible and anhedonia-resilient rats as compared with that of sham on day 7, 14, and 21 after SNI surgery (**Figure 1C**). However, there was no any change in the MWT between anhedonia-susceptible and anhedonia-resilient rats (**Figure 1C**). Furthermore, the sucrose preference in the anhedonia-susceptible rats was significantly lower than those in the sham or anhedonia-resilient rats on day 5, 12, and 19 after SNI (**Figure 1D**).

### Altered Expression of Keap1 and Nrf2 in Selected Tissues in Sham, Anhedonia-Susceptible, and Anhedonia-Resilient Rats

**FIGURE 2:**
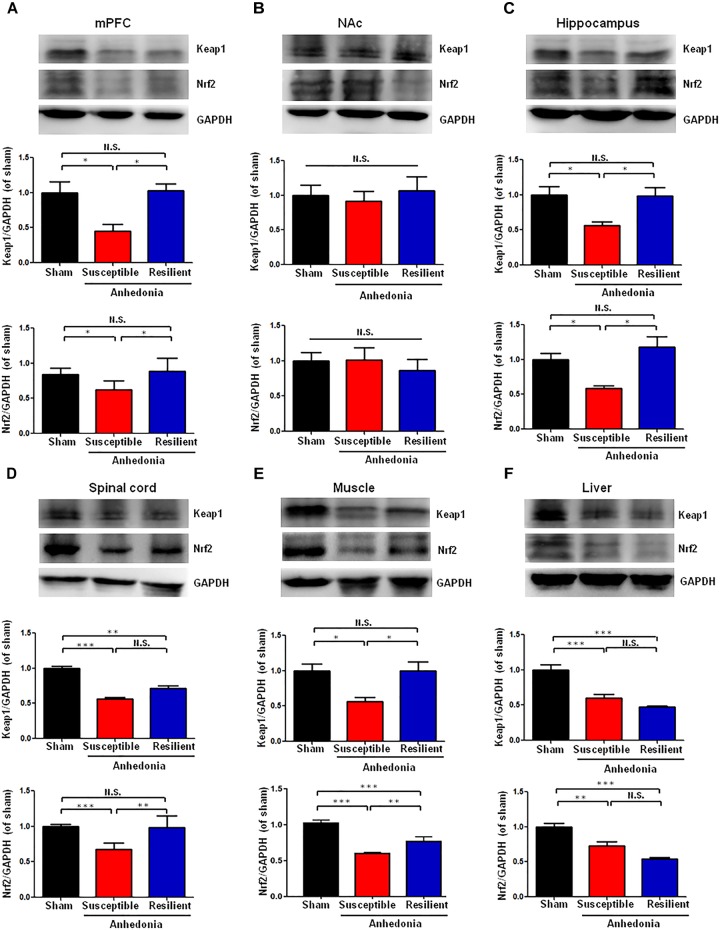
Expression of Keap1 and Nrf2 proteins in selected tissues among the sham, anhedonia-susceptible, and anhedonia-resilient groups. **(A)** Keap1 [*F*_(2,15)_ = 9.477_,_ *P* = 0.02] and Nrf2 [*F*_(2,15)_ = 11.497_,_ *P* = 0.013] levels in the mPFC. **(B)** Keap1 [*F*_(2,15)_ = 1.811, *P* = 0.243] and Nrf2 [*F*_(2,15)_ = 0.289, *P* = 0.759] levels in the NAc. **(C)** Keap1 [*F*_(2,15)_ = 10.833_,_ *P* = 0.01] and Nrf2 [*F*_(2,15)_ = 9.248_,_ *P* = 0.015] levels in the hippocampus. **(D)** Keap1 [*F*_(2,15)_ = 62.237_,_ *P* = 0.011] and Nrf2 [*F*_(2,15)_ = 31.095_,_ *P* < 0.001] levels in the L2-5 spinal cord. **(E)** Keap1 [*F*_(2,15)_ = 6.35_,_ *P* = 0.033] and Nrf2 [*F*_(2,15)_ = 144.8_,_ *P <* 0.001] levels in the muscle. **(F)** Keap1 [*F*_(2,15)_ = 25.886_,_ *P* = 0.001] and Nrf2 [*F*_(2,15)_ = 22.201_,_ *P* = 0.002] levels in the liver. Data are shown as mean ± SEM (*n* = 6). ^∗^*P* < 0.05, ^∗∗^*P* < 0.01, or ^∗∗∗^*P* < 0.01. Keap1, kelch-like ECH-associated protein 1; mPFC, medial prefrontal cortex; NAc, nucleus accumbens; Nrf2, nuclear factor (erythroid 2-derived)-like 2; N.S., not significant.

There were significant alterations in the levels of Keap1 protein in the mPFC, hippocampus, L2-5 spinal cord, muscle, and liver in the SNI-treated rats. *Post hoc* test showed a significant decrease of Keap1 protein in the mPFC, hippocampus and muscle in anhedonia-susceptible rats than that of sham and anhedonia-resilient rats (**Figures 2A,C,E**). Interestingly, there was a significant decrease of Keap1 protein in the L2-5 spinal cord and liver in anhedonia-susceptible rats than that of sham rats although there were no changes in the spinal cord and liver between anhedonia-susceptible and anhedonia-resilient rats (**Figures 2D,F**). In contrast, there were no changes of Keap1 in the NAc among the three groups (**Figure 2B**).

Anhedonia-susceptible rats showed a significant decrease of Nrf2 protein in the mPFC, hippocampus, spinal cord, and muscle compared to sham or anhedonia-resilient rats (**Figures 2A,C**). In contrast, the expression of Nrf2 protein in the NAc from anhedonia-susceptible rats was no difference among the three groups (**Figure 2B**). In the liver, both anhedonia-susceptible and anhedonia-resilient rats significantly decreased levels of Nrf2 compared to sham rats, but there was no significant change between the two groups (**Figure 2F**).

### Effects of Subsequent SFN Treatment on the Results of MWT and SPT After SNI Surgery

**FIGURE 3:**
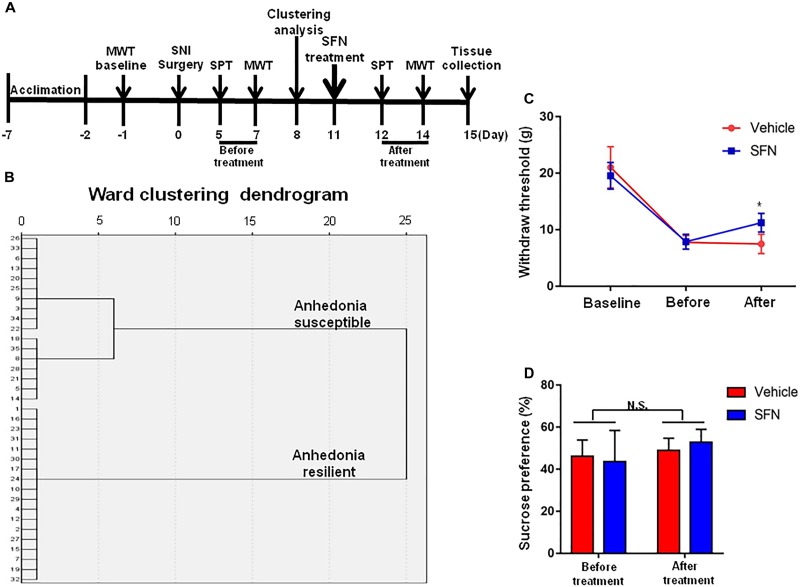
Effects of SFN treatment after SNI surgery on the results of MWT and SPT. **(A)** The schedule of the experiment. SNI surgery was performed on day 0 after acclimation. SFN (30 mg/kg, i.p.) was administered on day 11. SPT was measured on day 5 and 12 after SNI, respectively. MWT was performed on day 7 and 14 after SNI, respectively. **(B)** Dendrogram of hierarchical clustering analysis. A total of 35 SNI rats were divided into anhedonia-susceptible and anhedonia-resilient groups by SPT results. Thirteen-seven of 35 SNI rats were anhedonia-susceptible phenotype. **(C)** MWT [Time: *F*_(2,42)_ = 160.9_,_ *P* < 0.001; SFN: *F*_(1,42)_ = 1.635_,_ *P* = 0.208; Interaction: *F*_(2,42)_ = 6.284_,_ *P* = 0.0041]. Data are shown as mean ± SEM (*n* = 8). ^∗^*P* < 0.05, SFN group vs. vehicle group. **(D)** SPT [Time: *F*_(1,28)_ = 3.361_,_ *P* = 0.0774; SFN: *F*_(1,28)_ = 0.04322_,_ *P* = 0.8368; Interaction: *F*_(1,28)_ = 0.9291_,_ *P* = 0.3434]. Data are shown as mean ± SEM (*n* = 8). MWT, mechanical withdrawal test; N.S., not significant; SFN, sulforaphane; SNI, spared nerve injury; SPT, sucrose preference test.

Seventeen anhedonia-susceptible rats from 35 SNI rats were selected by hierarchical clustering analysis of SPT results (**Figures 3A,B**). The reduction of scores of MWT in anhedonia-susceptible rats after SNI surgery was attenuated significantly after subsequent single administration of SFN (30 mg/kg) (**Figure 3C**). In contrast, the reduced sucrose preference in anhedonia-susceptible rats after SNI surgery was not improved after subsequent single administration of SFN (**Figure 3D**).

### Effects of Subsequent SFN Treatment on the Levels of Keap1-Nrf2 Signaling in Selected Tissues After SNI Surgery

**FIGURE 4:**
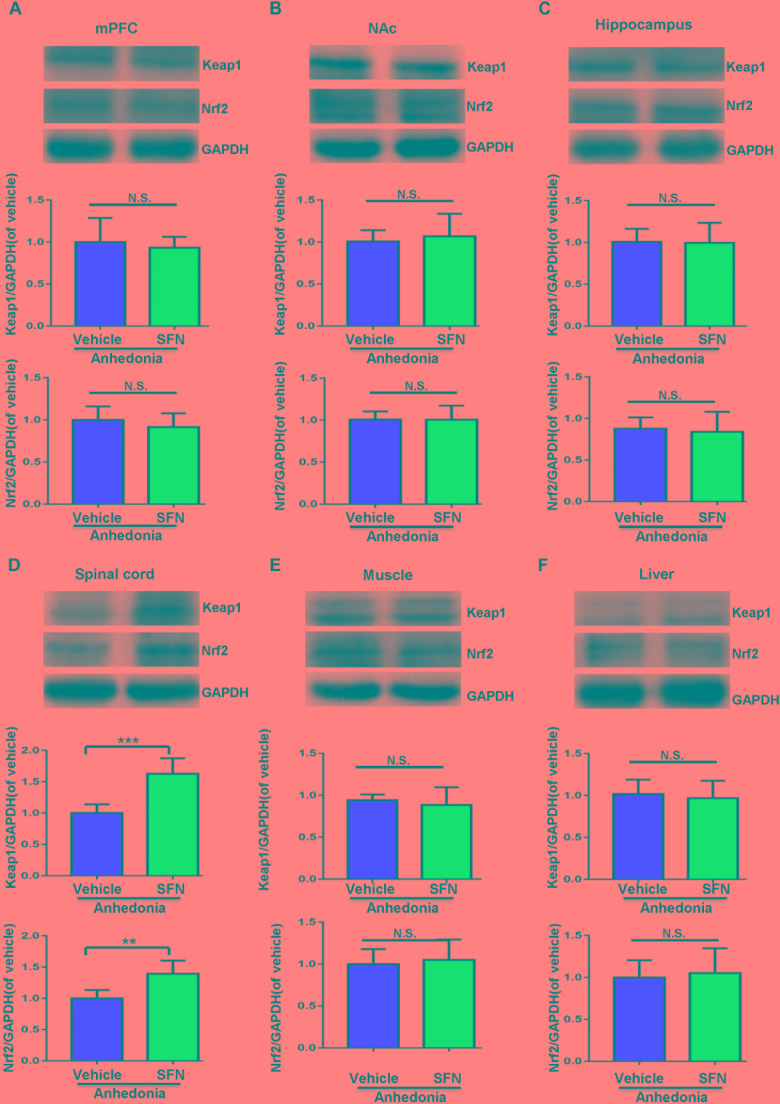
Effects of SFN treatment after SNI surgery on the expression of Keap1 and Nrf2 proteins in selected tissues. **(A)** Keap1 (*t* = 0.5248_,_ *P* = 0.6112) and Nrf2 (*t* = 0.9042_,_ *P* = 0.3872) levels in the mPFC. **(B)** Keap1 (*t* = 0.4853_,_ *P* = 0.6379) and Nrf2 (*t* = 0.01363_,_ *P* = 0.9894) levels in the NAc. **(C)** Keap1 (*t* = 0.1111_,_ *P* = 0.9137) and Nrf2 (*t* = 0.3427_,_ *P* = 0.7389) levels in the hippocampus. **(D)** Keap1 (*t* = 5.45_,_ *P* < 0.001) and Nrf2 (*t* = 3.861_,_ *P* = 0.0032) levels in the L2-5 spinal cord. **(E)** Keap1 (*t* = 0.6394_,_ *P* = 0.5369) and Nrf2 (*t* = 0.4178_,_ *P* = 0.6850) levels in the muscle. **(F)** Keap1 (*t* = 0.463_,_ *P* = 0.6533) and Nrf2 (*t* = 0.3559_,_ *P* = 0.7293) levels in the liver. Data are shown as mean ± SEM (*n* = 6). ^∗∗^*P* < 0.01 or ^∗∗∗^*P* < 0.001. Keap1, kelch-like ECH-associated protein 1; mPFC, medial prefrontal cortex; NAc, nucleus accumbens; Nrf2, nuclear factor (erythroid 2-derived)-like 2; N.S., not significant; SFN, sulforaphane.

A single administration of SFN (30 mg/kg) into anhedonia-susceptible rats after SNI surgery did not alter the levels of Keap1 and Nrf2 in the mPFC, NAc, hippocampus, muscle, and liver (**Figures 4A–C,E,F**). In contrast, SFN significantly increased the reduced levels of Keap1 and Nrf2 proteins in the spinal cord of anhedonia-susceptible rats (**Figure 4D**). Collectively, it is likely that activation of Keap1-Nrf2 system in the spinal cord by SFN may play a role in the SFN-induced beneficial effects for reduced MWT scores in anhedonia-susceptible rats after SNI.

### Effects of Pretreatment With SFN on the Results of MWT and SPT After SNI Surgery

**FIGURE 5:**
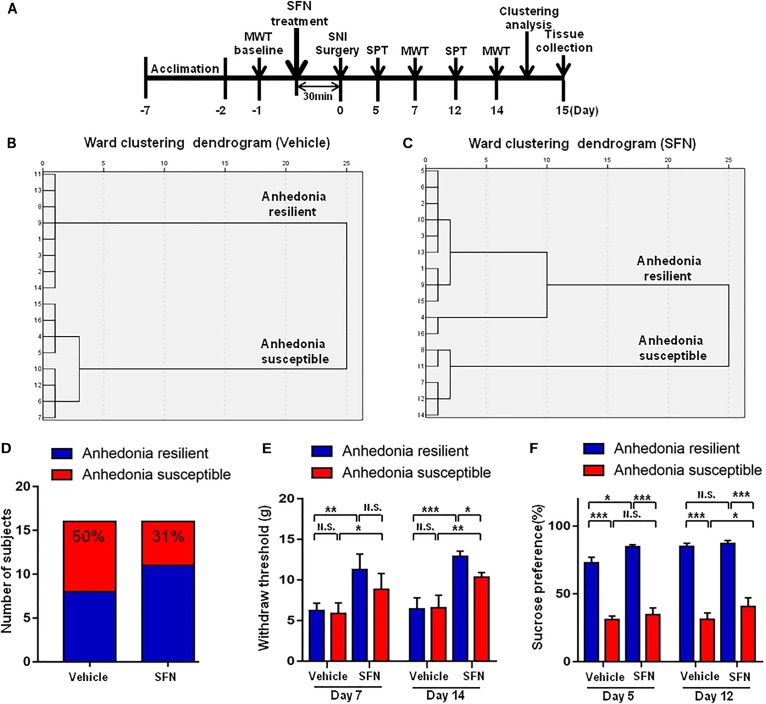
Effects of SFN treatment before SNI surgery on the results of MWT and SPT. **(A)** The schedule of this study. SNI was performed on day 0 after acclimation. SFN at a single dose of 30 mg/kg was administered 30 min before SNI surgery. MWT was measured on day 5 and 12 after SNI, respectively. SPT was performed on day 7 and 14 after SNI, respectively. **(B)** Dendrogram of hierarchical clustering analysis. A total of 16 SNI rats were divided into anhedonia-susceptible and anhedonia-resilient groups by SPT results. Eight of 16 SNI rats were anhedonia susceptible phenotype. **(C)** Dendrogram of hierarchical clustering analysis. A total of 16 SNI rats were divided into anhedonia-susceptible and anhedonia-resilient groups by SPT results. Five of 16 SNI rats were anhedonia-susceptible phenotype. **(D)** Ratio of anhedonia-susceptible rats to total SNI rats. The ratio of anhedonia-susceptible rats in vehicle and SFN groups were 50 and 31%, respectively. **(E)** MWT [Time: *F*_(1,16)_ = 8.851_,_ *P* = 0.0089; SFN: *F*_(3,16)_ = 30.58_,_ *P* < 0.001; Interaction: *F*_(3,16)_ = 0.9991_,_ *P* = 0.4186]. Data are shown as mean ± SEM (*n* = 5). ^∗^*P* < 0.05, ^∗∗^*P* < 0.01 or ^∗∗^*P* < 0.001. **(D)** SPT [Time: *F*_(1,16)_ = 32.05_,_ *P* < 0.001; SFN: *F*_(3,16)_ = 361.7_,_ *P* < 0.001; Interaction: *F*_(3,16)_ = 7.569_,_ *P* = 0.0023]. Data are shown as mean ± SEM (*n* = 5). MWT, mechanical withdrawal test; N.S., not significant; SFN, sulforaphane; SNI, spared nerve injury; SPT, sucrose preference test.

Spared nerve injury surgery was performed 30 min after a single administration of SFN (30 mg/kg) or vehicle (**Figure 4A**). The ratio of anhedonia-susceptible rats to total SNI rats in vehicle-treated group and SFN-treated group were 50 and 31%, respectively (**Figures 5B–D**). Thus, pretreatment with SFN significantly increased the number of anhedonia-resilient rats after SNI surgery (**Figure 5D**).

On day 7, SFN (30 mg/kg) significantly increased the scores of MWT in both anhedonia-susceptible and anhedonia-resilient rats (**Figure 5E**). On day 14, SFN-treated rats showed a significant increase in the MWT scores in both anhedonia-susceptible and anhedonia-resilient rats as compared with that of vehicle-treated rats. Furthermore, the MWT scores of SFN-treated anhedonia-resilient rats were significantly higher than those of SFN-treated anhedonia-susceptible rats (**Figure 5E**).

There was a significant change in the results of SPT between anhedonia-susceptible rats and anhedonia-resilient rats in both vehicle- and SFN-treated groups on day 5 and 12 (**Figure 5F**). On day 5, SFN significantly increased the sucrose preference of anhedonia-resilient rats, but not anhedonia-susceptible rats. On day 12, SFN significantly increased the sucrose preference of susceptible rats, but not resilient rats (**Figure 5F**).

### Prophylactic Effects of SFN on the Altered Levels of Keap1-Nrf2 Signaling in Selected Tissues After SNI Surgery

**FIGURE 6:**
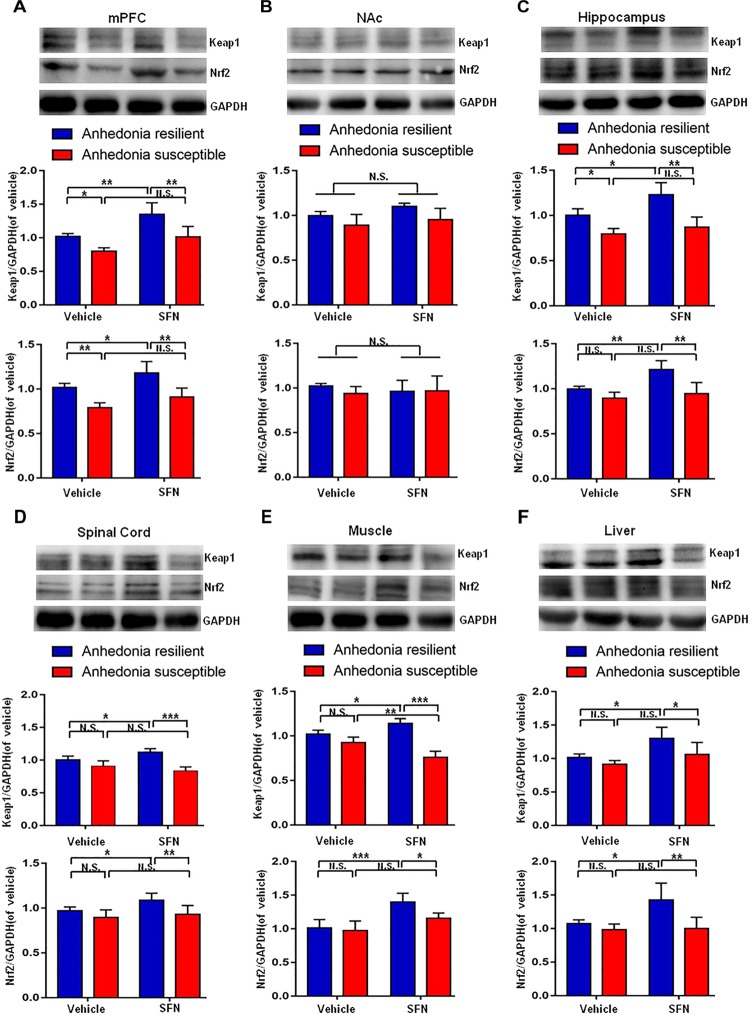
Effects of SFN treatment before SNI surgery on Keap1-Nrf2 signaling in selected tissues. **(A)** Keap1 [SFN: *F*_(1,16)_ = 25.1_,_ *P* = 0.001; Phenotype: *F*_(1,16)_ = 26.36_,_ *P* < 0.001; Interaction: *F*_(1,16)_ = 1.184_,_ *P* = 0.2926] and Nrf2 [SFN: *F*_(1,16)_ = 12.9_,_ *P* = 0.0024; Phenotype: *F*_(1,16)_ = 40.27_,_ *P* < 0.001; Interaction: *F*_(1,16)_ = 0.2745_,_ *P* = 0.6075] levels in the mPFC. **(B)** Keap1 [SFN: *F*_(1,16)_ = 3.965_,_ *P* = 0.0638; Phenotype: *F*_(1,16)_ = 9.697_,_ *P* = 0.0067; Interaction: F_(1,16)_ = 0.2142_,_ *P* = 0.6497] and Nrf2 [SFN: *F*_(1,16)_ = 0.088_,_ *P* = 0.7706; Phenotype: *F*_(1,16)_ = 0.6729_,_ *P* = 0.4241; Interaction: *F*_(1,16)_ = 0.8057_,_ *P* = 0.3827] levels in the NAc. **(C)** Keap1 [SFN: *F*_(1,16)_ = 11.79_,_ *P* = 0.0034; Phenotype: *F*_(1,16)_ = 41.42_,_ *P* < 0.001; Interaction: *F*_(1,16)_ = 2.94_,_ *P* = 0.1057] and Nrf2 [SFN: *F*_(1,16)_ = 12.01_,_ *P* = 0.0032; Phenotype: *F*_(1,16)_ = 23.12_,_ *P* < 0.001; Interaction: *F*_(1,16)_ = 4.539_,_ *P* = 0.049] levels in the hippocampus. **(D)** Keap1 [SFN: *F*_(1,16)_ = 20.69_,_ *P* < 0.001; Phenotype: *F*_(1,16)_ = 49.2_,_ *P* < 0.001; Interaction: *F*_(1,16)_ = 0.3084_,_ *P* = 0.5863] and Nrf2 [SFN: *F*_(1,16)_ = 13.11_,_ *P* = 0.0023; Phenotype: *F*_(1,16)_ = 34.32_,_ *P* < 0.001; Interaction: *F*_(1,16)_ = 1.179_,_ *P* = 0.2936] levels in the L2-5 spinal cord. **(E)** Keap1 [SFN: *F*_(1,16)_ = 4.55_,_ *P* = 0.0488; Phenotype: *F*_(1,16)_ = 291.3_,_ *P* < 0.001; Interaction: *F*_(1,16)_ = 149.2_,_ *P* < 0.001] and Nrf2 [SFN: *F*_(1,16)_ = 27.85_,_ *P* < 0.001; Phenotype: *F*_(1,16)_ = 6.673_,_ *P* = 0.02; Interaction: *F*_(1,16)_ = 3.469_,_ *P* = 0.081] levels in the muscle. **(F)** Keap1 [SFN: *F*_(1,16)_ = 14.27_,_ *P* = 0.0017; Phenotype: *F*_(1,16)_ = 9.022_,_ *P* = 0.0084; Interaction: *F*_(1,16)_ = 1.393, *P* = 0.2551] and Nrf2 [SFN: *F*_(1,16)_ = 6.814, *P* = 0.0189; Phenotype: *F*_(1,16)_ = 13.18_,_ *P* = 0.0023; Interaction: *F*_(1,16)_ = 5.598_,_ *P* = 0.0309] levels in the liver. Data are shown as mean ± SEM (*n* = 5). ^∗^*P* < 0.05, ^∗∗^*P* < 0.01, or ^∗∗∗^*P* < 0.001. Keap1, kelch-like ECH-associated protein 1; mPFC, medial prefrontal cortex; NAc, nucleus accumbens; Nrf2, nuclear factor (erythroid 2-derived)-like 2; N.S., not significant; SFN, sulforaphane.

Pretreatment with SFN (30 mg/kg) significantly increased the Keap1 and Nrf2 levels in the mPFC, hippocampus, spinal cord, muscle, and liver of anhedonia-resilient rats compared to vehicle-treated anhedonia-resilient rats or SFN-treated anhedonia-susceptible rats (**Figures 6A,C–F**). However, SFN did not induce any change in the levels of Keap1 and Nrf2 protein in the NAc from anhedonia-resilient rats and anhedonia-susceptible rats (**Figure 6B**).

## Discussion

The present study demonstrated that although SNI rats suffered almost identical nociceptive damage, some rats exhibited anhedonia-like phenotypes. Tissue levels of Keap1 and Nrf2 in mPFC, hippocampus, and muscle of rats with anhedonia-like phenotypes were lower than those in rats without anhedonia-like phenotypes and sham-operated rats. Furthermore, tissue levels of Keap1 and Nrf2 in the spinal cord and liver of rats with anhedonia-like phenotypes were lower than those of sham rats. Decreased MWT scores, but not decreased sucrose preference, in susceptible rats following SNI surgery were attenuated with subsequent single administration of SFN (30 mg/kg). Furthermore, SFN significantly improved the decreased Keap1 and Nrf2 expression levels in the spinal cord of susceptible rats following SNI. Moreover, SFN treatment (30 mg/kg) prior to SNI surgery significantly ameliorated the reduced MWT scores, anhedonia-like behavior, and decreased Keap1-Nrf2 signaling in mPFC, hippocampus, and spinal cord of rats with anhedonia-like phenotypes. To the best of our knowledge, this is the first study demonstrating the role of the Keap1-Nrf2 system in individual differences of anhedonia after neuropathic pain. In addition, this is the first study to establish the role of Keap1-Nrf2 signaling in the prophylactic and therapeutic effects of SFN in the comorbidity of neuropathic pain and depression in rodents.

In preclinical studies, depression-related behaviors of hyperalgesic rats were highly diverse. Using resident-intruder social interaction and sleep–wake analyses, it is reported that chronic constriction injury induced a subgroup (approximately 30%) of rats with altered dominant behavior (Monassi et al., 2003) and sleep–wake cycle (Austin and Moalem-Taylor, 2010). In the present study, hierarchical cluster analysis was used to divide SNI rats into two clusters: one group (approximately 68%; anhedonia-like phenotype) with reduced sucrose preference in the SPT and the other (approximately 32%; without anhedonia-like phenotype) with sucrose preference similar to that in sham-operated rats, consistent with our previous report (Fang et al., 2018). In the current study, we also found that rats, regardless of the presence or absence of anhedonia-like phenotype, exhibited similar MWT scores, suggesting that alterations in mood-related behaviors were independent of the degree of nociceptive damage, which was consistent with previous studies (Monassi et al., 2003; Austin and Moalem-Taylor, 2010; Austin et al., 2015; Gui et al., 2016; Xie et al., 2017; Fang et al., 2018).

We identified decreased Keap1-Nrf2 signaling in mPFC and hippocampus of rats with anhedonia-like phenotypes on day 23 following SNI. Low levels of Keap1-Nrf2 in mPFC and hippocampus are reportedly associated with the development of depression-like phenotypes, including anhedonia, in rodents (Yao et al., 2016a; Zhang et al., 2017). Furthermore, *Nrf2* knockout mice demonstrated depression-like phenotype, including anhedonia (Yao et al., 2016b). A recent study using postmortem brain samples exhibited reduced Keap1 and Nrf2 expression levels in the parietal cortex of depressed patients (Zhang et al., 2018), suggesting that decreased Keap1-Nrf2 signaling plays a role in depression. Interestingly, SFN did not improve the decreased sucrose preference in rats with anhedonia-like phenotypes, although it significantly attenuated decreased MWT scores through the improvement of the Keap1-Nrf2 system in the spinal cord of rats with anhedonia-like phenotype. Thus, it appears that Keap1-Nrf2 signaling in the spinal cord plays a role in pain following SNI. Overall, it is likely that decreased Keap1-Nrf2 signaling in mPFC and hippocampus is associated with anhedonia-like phenotype in rats with neuropathic pain, and that Nrf2 activators, including SFN, have therapeutic potential in patients with neuropathic pain-associated anhedonia.

In this study, we found that SNI rats with anhedonia-like phenotype had lower tissue Keap1 and Nrf2 levels in the spinal cord than those in sham-operated rats. Given the role of Keap1-Nrf2 signaling in pain (Kim et al., 2010; Redondo et al., 2017), reduced Keap1-Nrf2 signaling in the spinal cord may play a role in neuropathic pain, although not in anhedonia-like phenotypes.

Skeletal muscle, which consumes two-third of the body’s energy supply, comprises approximately 40% of the body mass in a healthy individual with normal body weight (Rolfe and Brown, 1997). Following skeletal muscle injury, Nrf2 activity is required for muscle regeneration and effective healing by regulating satellite cell proliferation (Shelar et al., 2016) and preventing inflammation-induced muscle wasting and fibrosis (Al-Sawaf et al., 2014). In the present study, we also found decreased Keap1 and Nrf2 expression levels in the muscle of anhedonia-susceptible rats. Furthermore, these levels were significantly lower than those in the muscle of anhedonia-resilient rats. Thus, decreased Keap1-Nrf2 signaling in the muscle may contribute to anhedonia susceptibility to SNI surgery, although further detailed studies are warranted.

In addition, we found decreased Keap1 and Nrf2 expression levels in the liver of rats with or without anhedonia-like phenotype following SNI surgery. Hence, it is unlikely that Keap1 and Nrf2 signaling alterations in the liver contribute to anhedonia susceptibility to SNI surgery. Given the role of the Keap1-Nrf2 system in the host cell defense against oxidative stress (Kobayashi et al., 2013; Suzuki et al., 2013; O’Connell and Hayes, 2015; Suzuki and Yamamoto, 2015; Wardyn et al., 2015; Cuadrado et al., 2018; Yamamoto et al., 2018), decreased Keap1-Nrf2 signaling in the liver may play a role in oxidative stress in the liver of SNI rats. Nonetheless, further detailed studies regarding the underlying role of Keap1-Nrf2 signaling in the brain and peripheral tissues (spinal cord, muscle, and liver) are needed.

In the present study, we used a single injection of SFN (30 mg/kg) in a rat SNI model. Our previous studies demonstrated that dietary intake of glucoraphanin (a precursor of SFN) can prevent the onset of behavioral abnormalities and biochemical changes in the brain following phencyclidine administration (Shirai et al., 2015), inflammation (Zhang et al., 2017), chronic social defeat stress (Yao et al., 2016a), and maternal immune activation (Matsuura et al., 2018). Collectively, dietary intake of glucoraphanin during the experimental period may show prophylactic effects in reducing MWT and SPT scores following SNI surgery. Therefore, further study on the long-term administration of SFN (or glucoraphanin) is of great interest. These results suggested that SFN or its precursor glucoraphanin could potentially be used for the treatment of anhedonia in patients with neuropathic pain, because they are naturally occurring compounds found in cruciferous vegetables. Finally, further study regarding the dietary intake of glucoraphanin-rich vegetables (or SFN supplements) in depressed patients with neuropathic pain is necessary to study their prophylactic effects.

## Conclusion

The current study suggests that decreased Keap1-Nrf2 signaling in mPFC, hippocampus, and muscle is associated with individual differences of the anhedonia-like phenotype in rats with neuropathic pain, whereas in the spinal cords of SNI rats, it is associated with neuropathic pain. Therefore, it is likely that Nrf2 activators, including SFN, are a potential therapeutic target for comorbid pain and anhedonia in patients with neuropathic pain.

## Author Contributions

CY, KH, and AL designed the study. SL, CY, XF, GZ, and NH performed the behavioral tests. SL, XF, and JG performed western blot. SL, CY, and KH drafted the manuscript. JG and HX revised the manuscript. All the authors approved the manuscript and submission.

## Conflict of Interest Statement

CY received the research support from B. Braun Medical Inc. The remaining authors declare that the research was conducted in the absence of any commercial or financial relationships that could be construed as a potential conflict of interest.
